# Prevalence of death due to pulmonary embolism after trauma

**DOI:** 10.4103/0970-2113.63610

**Published:** 2010

**Authors:** Rodrigo Florêncio Echeverria, André Luciano Baitello, José Maria Pereira de Godoy, Paulo César Espada, Rogério Yukio Morioka

**Affiliations:** *Department of Trauma, Medicine School of São José do Rio Preto, São Paulo, FAMERP, Brazil*; 1 *Department of Cardiology and Cardiovascular, Medicine School of São José do Rio Preto, São Paulo, FAMERP, Brazil*

**Keywords:** *Causa mortis*, trauma, pulmonary embolism, prevalence, mortality

## Abstract

**Background:**

Pulmonary thromboembolism is an important cause of death affecting thousands of people worldwide. The current study aims to evaluate the prevalence of death due to pulmonary embolism after trauma.

**Materials and Methods:**

The diagnoses of the *causa mortis* of all patients treated in the Accident and Emergency Department of Hospital de Base in São José do Rio Preto, in the period from July 2004 to June 2005, were identified from autopsy reports to check whether pulmonary thromboembolism was involved.

**Result:**

A total of 109 deaths due to trauma were detected in this period with pulmonary embolism occurring in 3 (2.75%) patients.

**Conclusion:**

Pulmonary thromboembolism is an important cause of mortality in trauma patients and so prophylactic measures should be taken during the treatment of these patients.

## INTRODUCTION

The trauma population is at increased risk of venous thromboembolic disease, a potentially preventable cause of mortality and morbidity. Venous thromboembolism (VTE) remains an area of active clinical research focusing on evolving diagnostic techniques, newer methods of chemical and mechanical prophylaxis and improved understanding of the etiological factors of posttraumatic VTE.[1]

Studies show six risk factors to be independently significant by multivariate logistic regression for VTE were age ≥ 40 years, lower limb fractures with abbreviated injury scale (AIS) ≥ 3, head injury with AIS ≥ 3, ventilator days > 3, venous injury and a major operative procedure.[2] However, post-trauma death rates due to pulmonary embolism have not been explored. The objective of the current study was to evaluate the prevalence of death due to pulmonary embolism in accident patients.

## MATERIALS AND METHODS

The diagnoses of *causa mortis* of consecutive patients, who died in the Accident and Emergency Department of Hospital de Base in São José do Rio Preto, in the period from July 2004 to June 2005, were investigated to identify all patients who died due to pulmonary thromboembolism. Diagnoses were obtained from autopsy reports for all fatal accident victims in this period. Percentages were utilized for statistical analysis.

## RESULTS

In the period from July 2004 to June 2005, 109 deaths occurred due to accidents, with pulmonary embolism being the cause in three (2.75%). The types of accidents were: automobile collisions (22), motorcycle crashes (11), pedestrian-vehicle collisions (18), falls (22), bicycle accidents (4), gunshot injuries (18), stabbings (5), fights (6), burns (1), electrical shock (1) and unidentified injury (1).

Of these patients, 45 suffered cardiorespiratory arrest at admission, 11 had systolic pressure < 90 mmHg and 54 had systolic pressure > 90 mmHg. Sixty-four patients died on the day 1 of hospitalization, 38 between the day 2 to 10, five between day 11 29 and the remaining two patients died after the day 29. More than 50% of the patients arrived at hospital with a Glasgow scale score of 3 [Table 1]. Sixty-nine per cent of the patients lost consciousness at the site of the accident, 64% suffered blunt traumas and 87% underwent surgery.

**Table 1:** Evaluation of the Glasgow scale at admittance

Glasgow scale	3	4	6	7	8	9	10	11	12	13	14	15
No. of patients	64	5	2	1	2	1	2	2	2	3	3	24

The other causes of death included sepsis (5 patients), traumatic brain injuries (45), hemorrhagic shock (48), pneumonia (5), and electrocution (1) and for two patients the cause was not determined. Almost all the patients who suffered from hemorrhagic shock died on the day of the accident [Table 2]. Table 2 shows the patients grouped according to the type of trauma: head injury, injuries of other regions and hemorrhagic shock and the time of hospitalization before death.

**Table 2:** Time of hospitalization of patients with head injury, unspecific injuries and hemorrhagic shock

Hospital stay	1 day	2-10 days	11-29 days	>30 days
General trauma	58	35	5	2
Head injuries	46	49	5	0
Hemorrhagic shock	97	3	0	0

Values are in percentage

## DISCUSSION

The current study shows the prevalence of death due to pulmonary embolism in an Accident and Emergency Department in Brazil. These data show the importance of pulmonary thromboembolism as the cause of death in accident victims. The literature suggests that chemical and mechanical prophylactic techniques, whether associated or not, may reduce this rate.[3‐7] There is no consensus with respect to the advantages of the different methods of prophylaxis. The risks and difficulties of the different methods have identified specific types of injuries that benefit more from one determined prophylactic option. This is the case of head injuries, where mechanical methods may represent a lower risk for patients. The identification of risk factors for thromboembolism helps identify individuals for whom prophylaxis would be advantageous.

Mortality due to thromboembolism, identified in the current study, shows that Accident and Emergency Departments should define a prophylactic conduct with the aim of reducing this cause of death.

African-American patients have a significantly higher rate of VTE, particularly following exposure to a provoking risk factor such as surgery, medical illness or trauma. In addition, African-Americans are more likely to be diagnosed with pulmonary embolism (PE) than deep-vein thrombosis (DVT) compared to Caucasians and other racial groups.[8]

The incidence of DVT among injured patients with traumatic brain injury is significantly higher than those patients without head injury, independent of anticoagulation therapy. Rigorous surveillance to detect DVT among trauma patients with traumatic brain injury should be undertaken and appropriate alternate means for the prevention of pulmonary thromboembolism used.[9] The rate of VTE at trauma centers is high, which is expected given the complexity of patients treated and higher ISS. Patients with an ISS greater than 15, need for operation, spinal cord injuries, lower extremity fractures, and certain thoracic injuries are at risk for VTE.[10] In one study pulmonary embolism was silent in 63% and DVT was asymptomatic in 68% of patients.[11]

In our study, some aspects such as the loss of consciousness at the site of the accident, seen in 69% of the patients who died - a significant marker of death, called for our attention. All the patients who were admitted to hospital suffering from cardiorespiratory arrest were submitted to cardiopulmonary resuscitation, however, all of them died. This is another important risk factor for death after trauma. The patients with pulmonary embolism as the *causa mortis* died after the second day, and thus death may be associated with trauma and immobilization. These patients presented with two of the three well characterized risk factors of Virchow's triad, that is, trauma and immobilization. The prevalence of DVT in patients with leg bones fractured during accidents is about 12%.[12,13]

## CONCLUSION

Pulmonary thromboembolism influences the prognosis of trauma victims and is even one cause of death. This highlights the importance of preventive measures during the treatment of these patients.

## References

[1] Knudson MM, Ikossi DG (2004). Venous thromboembolism after trauma. Curr Opin Crit Care.

[2] Velmahos GC, Toutouzas KG, Brown C, Vassiliu P, Gkiokas G, Rhee P (2004). Thromboprophylaxis does not protect severely injured patients against pulmonary embolism. Am Surg.

[3] Knudson MM, Morabito D, Paiement GD, Shackleford S (1996). Use of low molecular weight heparin in preventing thromboembolism in trauma patients. J Trauma.

[4] Elliott CG, Dudney TM, Egger M, Orme JF, Clemmer TP, Horn SD (1999). Calf-thigh sequential pneumatic compression compared with plantar venous pneumatic compression to prevent deep-vein thrombosis after non-lower extremity trauma. J Trauma.

[5] Nathens AB, McMurray MK, Cuschieri J, Durr EA, Moore EE, Bankey PE (2007). The practice of venous thromboembolism prophylaxis in the major trauma patient. J Trauma.

[6] Cothren CC, Smith WR, Moore EE, Morgan SJ (2007). Utility of once-daily dose of low-molecular-weight heparin to prevent venous thromboembolism in multisystem trauma patients. World J Surg.

[7] Limpus A, Chaboyer W, McDonald E, Thalib L (2006). Mechanical thromboprophylaxis in critically ill patients: A systematic review and meta-analysis. Am J Crit Care.

[8] White RH, Keenan CR (2009). Effects of race and ethnicity on the incidence of venous thromboembolism. Thromb Res.

[9] Reiff DA, Haricharan RN, Bullington NM, Griffin RL, McGwin G, Rue LW 3rd (2009). Traumatic brain injury is associated with the development of deep vein thrombosis independent of pharmacological prophylaxis. J Trauma.

[10] Azu MC, McCormack JE, Huang EC, Lee TK, Shapiro MJ (2007). Venous thromboembolic events in hospitalized trauma patients. Am Surg.

[11] Sharma OP, Oswanski MF, Joseph RJ, Tonui P, Westrick L, Raj SS (2007). Venous thromboembolism in trauma patients. Am Surg.

[12] Baitelo AL, Palcheti JC, Coraçari AR, Figueredo RA, Lima FA, Godoy JM (2001). Rastreamento da trombose venosa profunda em pacientes politraumatizados com fratura de membros inferiores. HB Científica.

[13] Lu Y, Ma B, Guo R, Wang Y, Zhang J, Wu Y (2007). Deep vein thrombosis in trauma: A prospective study of lower limb orthopedic trauma patients in Tianjin Hospital, China. Int Angiol.

